# A Combinatorial Relative Mass Value Evaluation of Endogenous Bioactive Proteins in Three-Dimensional Cultured Nucleus Pulposus Cells of Herniated Intervertebral Discs: Identification of Potential Target Proteins for Gene Therapeutic Approaches

**DOI:** 10.1371/journal.pone.0081467

**Published:** 2013-11-21

**Authors:** Demissew S. Mern, Johann Fontana, Anja Beierfuß, Claudius Thomé, Aldemar A. Hegewald

**Affiliations:** 1 Department of Neurosurgery, Innsbruck Medical University, Innsbruck, Austria; 2 Department of Neurosurgery, University Medical Center Mannheim, Heidelberg University, Mannheim, Germany; Centro de Investigacion y de Estudios Avanzados del Instituto Politecnico Nacional, Mexico

## Abstract

Painful degenerative disc diseases have been targeted by different biological treatment approaches. Nucleus pulposus (NP) cells play a central role in intervertebral disc (IVD) maintenance by orchestrating catabolic, anabolic and inflammatory factors that affect the extracellular matrix. IVD degeneration is associated with imbalances of these factors, resulting in a catabolic inflammatory metabolism. Therefore, accurate knowledge about their quantity and quality with regard to matrix synthesis is vital for a rational gene therapeutic approach. NP cells were isolated from 63 patients operated due to lumbar disc herniation (mean age 56 / range 29 - 84 years). Then, three-dimensional culture with low-glucose was completed in a collagen type I scaffold for four weeks. Subsequently cell proliferation evaluation was performed using 3-(4, 5-dimethylthiazolyl-2)-2,5-diphenyltetrazolium bromide and intracellular concentration of 28 endogenously expressed anabolic, catabolic, inflammatory factors and relevant matrix proteins was determined by enzyme-linked immunosorbent assay. Specimen-related grades of degeneration were confirmed by preoperative magnetic resonance imaging. Independent from gender, age and grade of degeneration proliferation rates remained similar in all groups of NP cells. Progressive grades of degeneration, however, showed a significant influence on accumulation of selective groups of factors such as disintegrin and metalloproteinase with thrombospondin motifs 4 and 5, matrix metalloproteinase 3, metalloproteinase inhibitor 1 and 2, interleukin-1β and interleukin-1 receptor. Along with these changes, the key NP matrix proteins aggrecan and collagen II decreased significantly. The concentration of anabolic factors bone morphogenetic proteins 2, 4, 6 and 7, insulin-like growth factor 1, transforming growth factor beta 1 and 3, however, remained below the minimal detectable quantities. These findings indicate that progressive degenerative changes in NP may be problematic with regard to biologic treatment strategies. Hence, gene therapeutic interventions regulating relevant bioactive factors identified in this work might contribute to the development of regenerative treatment approaches for degenerative disc diseases.

## Introduction

Painful degenerative disc diseases, frequently caused by genetic predisposition, have been targeted by different biological treatment approaches [1,2]. These include the administration of growth factors, the application of autologous or allogenic cells, gene therapy, in situ therapy and the introduction of biomaterials or a combination thereof. 

Nucleus pulposus (NP) cells play a central role in intervertebral disc (IVD) matrix maintenance by orchestrating several catabolic, anabolic and inflammatory factors that affect the extracellular matrix [3,4]. IVD degeneration is associated with imbalances of these factors, resulting in a catabolic inflammatory metabolism. Consequently, accurate knowledge about their quantity as well as their quality with regard to matrix synthesis is vital for a rational gene therapeutic approach. In the current experimental literature, the number of cells and the concentration of gene therapeutic factors applied for regeneration of NP in animal models fluctuate in part enormously, which indicates a deficit in data about cell proliferation rates and concentrations of target proteins in NP cells [5-11].

With this work, we intended a screening for the identification of potential target proteins for gene therapeutic approaches in a tissue engineering setting with human nucleus pulposus cells in a three-dimensional collagen type I scaffold. Therefore, we analyzed cell proliferation and intracellular protein concentration of 28 endogenously expressed anabolic, catabolic and inflammatory factors as well as matrix proteins in relation with age, gender and grade of degeneration by using 3-(4, 5-dimethylthiazolyl-2)-2,5-diphenyltetrazolium bromide (MTT) assay and enzyme-linked immunosorbent assay (ELISA) respectively. Specimen-related grades of degeneration were confirmed by preoperative magnetic resonance imaging.

Previous data on expressions profiles of NP cells were determined either qualitatively by histological and immunohistological methods or quantitatively by RNA techniques [12-17].

The advantage of the relative mass value evaluation method is the ability to perform precise quantifications of protein concentrations. To our knowledge this method has not been applied to analyze a broad array of endogenously expressed bioactive factors in NP cells from a larger number of specimens.

Here, we determined the relative mass value of 28 endogenously expressed target protein in NP cells isolated from 63 tissue specimens and analysed the unfavorable phenotypic alternations that might restrain NP matrix regeneration. Progressive grades of degeneration, showed a significant influence on accumulation of selective bioactive factors. The results of this study might contribute to the development of regenerative treatment approaches for degenerative disc diseases.

## Materials and Methods

### Tissue samples

**Figure 1:**
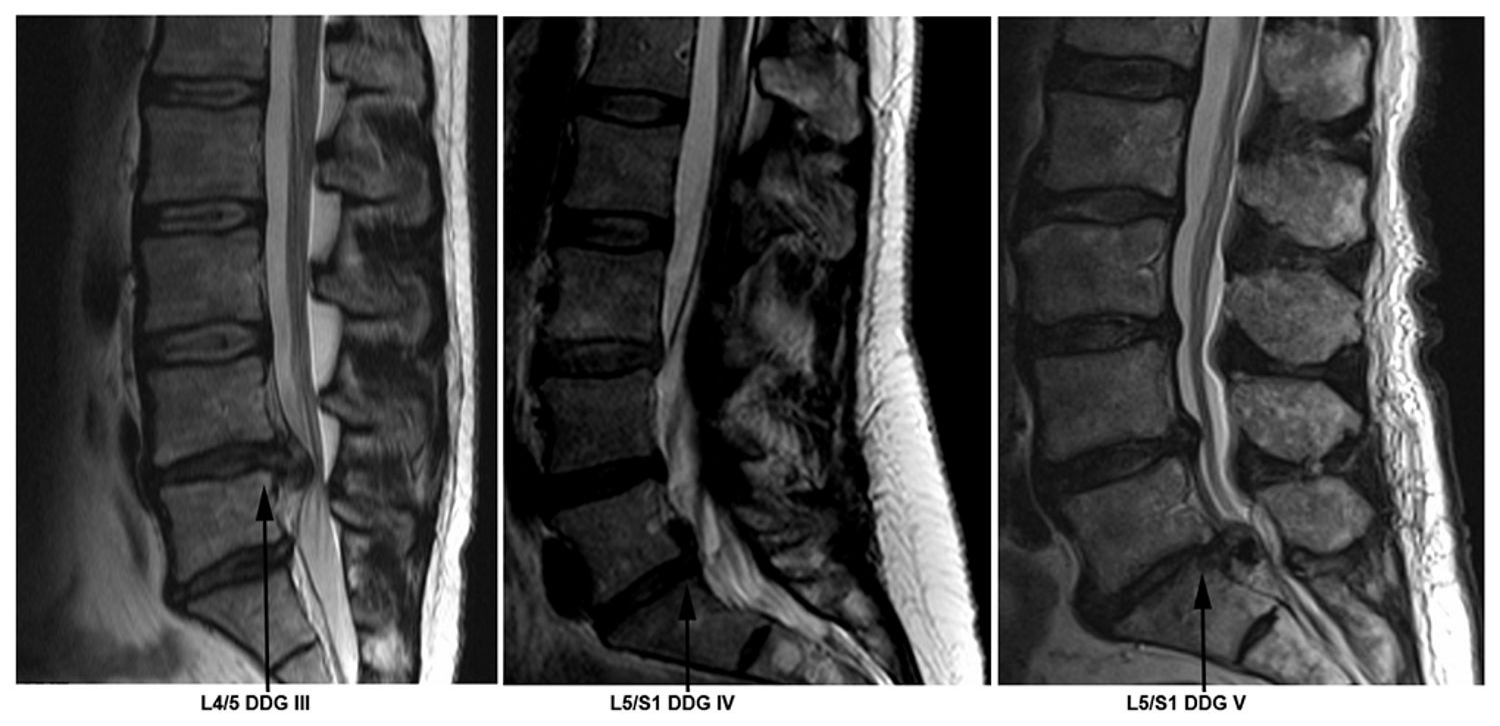
MRI showing representative images of disc degeneration grades (DDG) III, IV and V with lumbar disc herniation.

Experimental studies of human spinal disc specimens were approved by the local research ethics committee (Heidelberg University, University Medical Center Mannheim: project 2009-217N-MA). Human NP tissues were obtained during surgery with informed consents of the patients. Participants provided their written informed consent to participate in this study. Scoring of spinal disc degeneration was based on magnetic resonance imaging (MRI) findings according to the Pfirrmann scoring system [18]. Representative images of disc degeneration grades III, IV, and V with lumbar disc herniation are shown in Figure 1. The study involved 63 lumbar discs of degeneration grade III, IV and V with a mean age of 56 years (range 29 - 84 years, 27 women and 36 men). More information is presented in table 1. Patient NP specimens were recruited from NP compartment during surgical procedure conducted on individuals with intervertebral disc herniation. Inclusion criteria for surgery were radiographically determined intervertebral disc herniations with nerve root compression on MRI, which correlated to primary symptoms that remained unresponsive to non-operative treatment for six weeks or demonstrated progressive neurological deterioration in the face of conservative treatment. NP specimens were immediately brought to the lab in sterile phosphate buffered saline solution (PBS) (Sigma-Aldrich) for instant cell isolation.

**Table 1:** Details of NP samples with disc level, disc degeneration grade (DDG), age and gender.

**Sample**	**Disc level**	**DDG**	**Year/Sex**		**Sample**	**Disc level**	**DDG**	**Year/Sex**
1	L4/5	III	29/F		33	L5/S1	IV	47/M
2	L4/5	III	30/M		34	L3/4	IV	49/F
3	L5/S1	III	32/M		35	L4/5	IV	57/M
4	L4/5	III	33/F		36	L4/5	IV	58/M
5	L4/5	III	36/F		37	L5/S1	IV	59/M
6	L5/S1	III	37/M		38	L3/4	IV	61/F
7	L5/S1	III	39/F		39	L4/5	IV	67/F
8	L5/S1	III	39/F		40	L4/5	IV	71/M
9	L4/5	III	42/M		41	L4/5	IV	73/F
10	L4/5	III	44/F		42	L4/5	V	61/F
11	L5/S1	III	44/F		43	L4/5	V	62/M
12	L5/S1	III	45/M		44	L4/5	V	63/F
13	L4/5	III	47/M		45	L4/5	V	65/M
14	L4/5	III	47/M		46	L5/S1	V	66/F
15	L5/S1	III	49/M		47	L4/5	V	66/M
16	L5/S1	III	50/F		48	L3/4	V	67/M
17	L5/S1	III	51/M		49	L4/5	V	67/M
18	L5/S1	III	52/M		50	L5/S1	V	67/M
19	L5/S1	III	52/F		51	L5/S1	V	67/M
20	L5/S1	III	54/M		52	L5/S1	V	67/F
21	L4/5	III	54/M		53	L4/5	V	69/F
22	L3/4	III	56/M		54	L5/S1	V	69/F
23	L5/S1	III	59/M		55	L5/S1	V	71/M
24	L4/5	III	62/M		56	L4/5	V	72/F
25	L2/3	III	67/M		57	L2/3	V	72/F
26	L4/5	III	76/M		58	L3/4	V	74/M
27	L5/S1	IV	32/F		59	L5/S1	V	79/M
28	L5/S1	IV	41/M		60	L4/5	V	79/F
29	L5/S1	IV	43/M		61	L5/S1	V	79/F
30	L3/4	IV	44/M		62	L5/S1	V	84/F
31	L5/S1	IV	44/F		63	L4/5	V	84/F
32	L 4/5	IV	44/M					

Samples from NP compartment were obtained from patients undergoing surgery due to spinal disc herniation.

### Isolation and monolayer expansion of NP cells

IVD tissues were washed twice in PBS (1000 x g, 2 min) and NP tissues were carefully separated from AF (anulus fibrosus) on the basis of their macroscopic morphology (identification of the innermost lamellar ring of the AF) and finely minced into small fragments of approximately 2 mm^3^. Samples were directly digested with 0.02 % w/v pronase (Sigma-Aldrich) (1 h, 37 °C, 5 % CO_2_) in 20 ml DMEM (Dulbecco's Modified Eagle's Medium) containing 1 % penicillin/streptomycin, 1 % glucose and 1 % FCS (fetal calf serum) (Sigma-Aldrich). Samples were filtered through sterile 75 gm nylon mesh filters (Sigma-Aldrich) and supernatants were centrifuged (1000 x g, 2 min). Pellets were resuspended again in 20 ml DMEM and digested with 0.02 % w/v collagenase II (Sigma-Aldrich) and 100U hyaluronidase (Sigma-Aldrich) (3 h, 37 °C, 5 % CO_2_). Samples were filtered through sterile 75 gm nylon mesh filters and supernatants were centrifuged (1000 x g, 2 min). Pellets were resuspended in 10 ml DMEM containing 1 % penicillin/streptomycin, 1 % glucose and 10 % FCS and cultured in 75 cm^2^ tissue culture flask (Sigma-Aldrich) (2 weeks, 37 °C, 5 % CO_2_) by changing the culture medium every two days. Cells were cryopreserved at -196 °C in culture medium containing 30 % FCS and 15 % dimethyl sulfoxide (DMSO) (Sigma-Aldrich).

### Three dimensional NP cell culture in collagen I scaffold

The scaffold collagen cell carrier (CCC) (Viscofan Bioengineering) was used for three dimensional (3D) cell cultures. Each well of the 6-well plates was loaded with 1.5 ml PBS (pH 7.3 without Ca^2+^ / Mg^2+^) to attach the 6-well format scaffold to the bottoms. Each scaffold was placed on to the PBS and incubated at room temperature for 20 min. After removing the remaining PBS, the plates were left in the operating laminar flow hood overnight. Prior to cell seeding, the scaffold was equilibrated by incubation with 1.5 ml pre-warmed culture medium (10 min, 37 °C, 5 % CO_2_). The medium was removed and an aliquot (4 x 10^5^ cells/1.5 ml) of the cells was seeded onto the scaffold. Cells were cultured (37 °C, 5 % CO_2_) by changing the culture medium every two days and harvested after four weeks for analysis of cell proliferation and protein expression.

### Isolation of NP cells from collagen I scaffold

Following four weeks of 3D cell culture, the scaffold was digested in 1 ml culture medium with 0.02 % w/v collagenase II (1 h, 37 °C, 5 % CO_2_). Samples were filtered through sterile 75 gm nylon mesh filters and supernatants were centrifuged (1000 x g, 2 min). Pellets were washed twice in 5 ml PBS (1000 x g, 2 min) and processed for assays of cell proliferation and protein expression.

### NP cell proliferation assay

After culturing of 4 x 10^5^ cells in the scaffold for four weeks, cell proliferation assay was determined by using 3-(4, 5-dimethylthiazolyl-2)-2,5-diphenyltetrazolium bromide (MTT) (Molecular Probes). NP cells from the scaffold were suspended in 0.5 ml culture medium and duplicates of 100 µl were plated into flat-bottomed 96 well plates. Duplicate control wells of medium alone were added to provide the blanks for absorbance readings. Cells were incubated (24 h, 37 °C, 5 % CO_2_) to recover from handling. After adding 10 µl MTT Reagent to each well, cells were again incubated (3 h, 37 °C, 5 % CO_2_) and 100 µl of the SDS-HCl solution was added for further incubation (4 h, 37 °C, 5 % CO_2_). The absorbance in each well was measured at 570 nm in a microtiter plate reader Infinite 200 (TECAN). The average value of the blank duplicate readings was subtracted from the average values of the sample duplicate readings and cell concentration was calculated from the standard curve.

### Isolation and quantification of protein from NP cells

Protein isolation was performed with NP cells harvested from the scaffold using radio-immunoprecipitation assay (RIPA) buffer (Sigma-Aldrich). Cell pellets were washed twice in cold PBS (2,500 x g, 5 min), resuspended with 300 ml cold RIPA buffer containing protease inhibitor and phosphatase inhibitor cocktails (Sigma-Aldrich) and sonicated (30 sec, 50 % pulse). The Mixture was shacked gently on ice (15 min) and centrifuged (14,000 x g, 4 °C and 15 min). Supernatants were transferred to new tubes for protein quantification. Determination of protein concentration in samples was performed according to the instruction manual (Pierce Micro BCA Protein Assay Protocol) (Thermo Scientific).

### Enzyme-linked immunosorbent assay

Enzyme-linked immunosorbent assay (ELISA) was applied to determine the presence and concentration of target proteins in 100 µg of total protein extracts of NP cells. For measuring the concentration of target proteins ELISA kits were purchased from kits producers R & D Systems (United Kingdom) or Uscn Life Science Inc. (USA). Assays were performed according to the instruction manuals of kits producers. The investigated 28 target proteins include: catabolic factors ADAMTS-4, ADAMTS-5 (a disintegrin and metalloproteinase with thrombospondin motifs), MMP-1, MMP-2, MMP-3, MMP-7, MMP-8, MMP-9, MMP-10 and MMP-13 (matrix metalloproteinase), anti-catabolic factors TIMP-1, TIMP-2, TIMP-3 and TIMP-4 (metalloproteinase inhibitor), inflammatory cytokines IL-1β (interleukin-1β), IL-1 R1 (interleukin-1 receptor), TNF- α (tumornekrosefaktor-α) and TNF-R1(tumornekrosefaktor receptor R1); anabolic factors BMP-2, BMP-4, BMP-6, BMP-7 (bone morphogenetic proteins), IGF-1 (insulin-like growth factor 1), TGF-β1 and TGF-β3 (transforming growth factor betas) as well as matrix proteins aggrecan, collagen I and collagen II.

### Statistical data analysis

The reliability of the MRI evaluations was predicted using agreement percentage and kappa statistics between two raters (interobserver reliability), and frequency of disagreement was calculated for each degeneration grade [18,19]. Statistical analysis was performed using GraphPad Prism version 6.0b for Mac OS X, GraphPad Software, La Jolla California USA, www.graphpad.com. For cell proliferation and protein expression levels 1-way ANOVA with Brown-Forsythe test and multiple comparisons were applied. Cell proliferation rates and concentration of target proteins were analyzed separately for each experiment. Correlation analysis was performed using the software IBM SPSS Statistics 20, Armonk New York USA to calculate the significance of the changes in cell proliferation and protein concentration as a function of degeneration grade, age and gender. Significance in all cases was set at P < 0.05.

Using the correspondence address all raw data are available at the Department of Neurosurgery, Innsbruck Medical University, Anichstrasse 35, A-6020 Innsbruck, Austria.

## Results

### Interobserver reliability

Interobserver reliability agreement for rating intervertebral disc degeneration with two observers was rated with a reliability coefficient kappa of 0.9031 and the calculated frequency of disagreements for Pfirrmann score of degeneration grade III, IV and V were 9.68 %, 15.00 % and 0.00 % respectively

### Proliferation rates of degenerative NP cells

**Figure 2:**
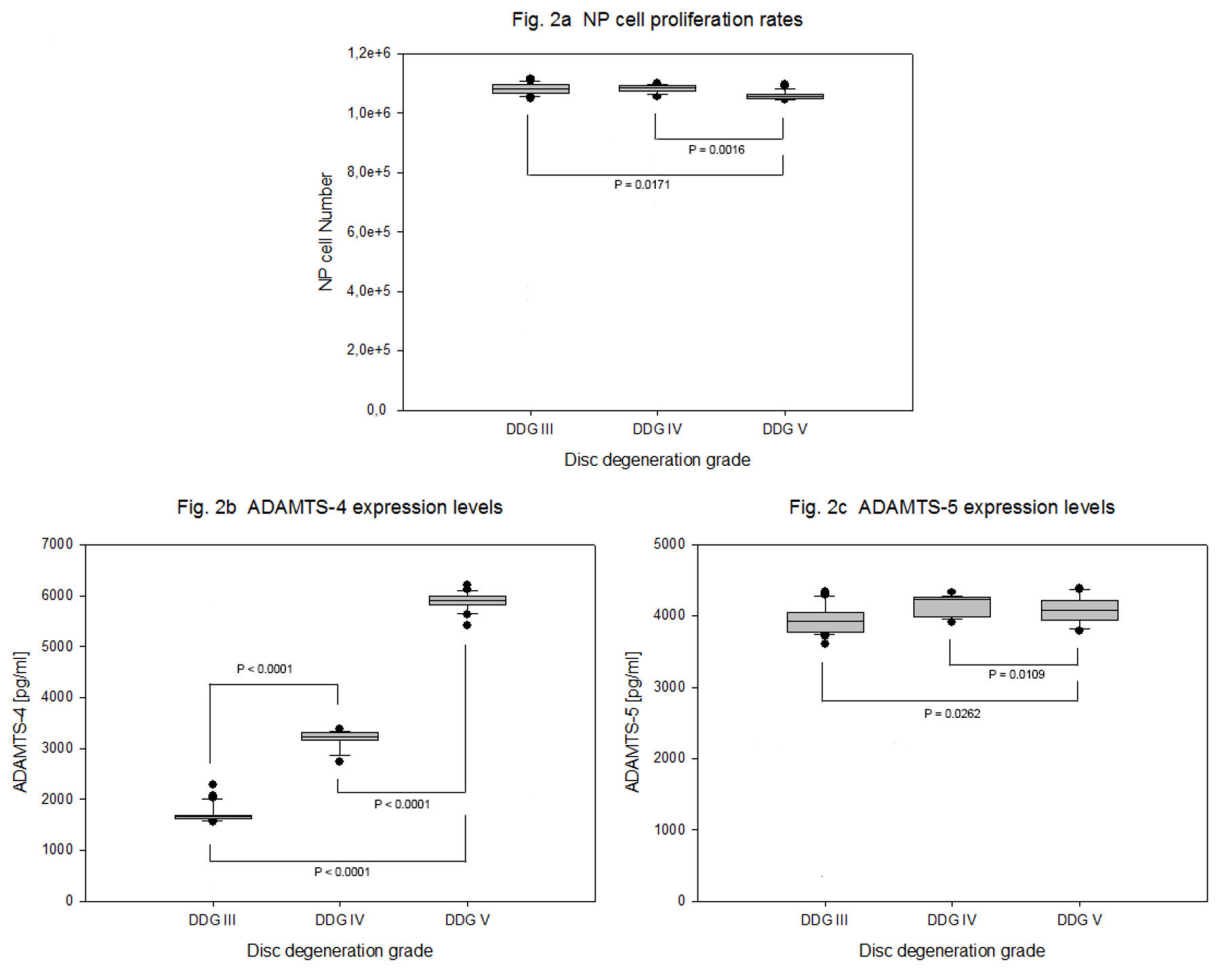
Proliferation rate of adult NP cells and expression levels of ADAMTS-4 and -5. NP cells were isolated from 63 samples of degenerative IVDs and 4 x 10^5^ cells from each sample were grown for four weeks in collagen I scaffold. Cell proliferation data (MTT assay) and data of ADAMTS-4 and -5 protein concentration (ELISA) from 100 µg total protein extracts were statistically analyzed on the bases of disc degeneration grade (DDG). Box plots with whiskers min. to max. show NP cell proliferation rates (Figure 2a), ADAMTS-4 protein expression levels (Figure 2b) and ADAMTS-5 protein expression levels (Figure 2c).

Three dimensional culture of 4 x 10^5^ adult NP cell in collagen I scaffold for four weeks resulted in similar proliferation rates for all tested samples of degeneration grade III, IV and V with mean values of 1,081 x 10^6^ (+ 19182), 1,083 x 10^6^ (+ 13121) and 1,056 x 10^6^ (+ 13857) cells respectively (P = 0.0014). Only about 2 % growth rate difference was confirmed between degeneration grade III - V (table 2 and Figure 2a). Neither age nor gender appears to play a role in influencing cell proliferation potential in adult NP (data not shown).

**Table 2:** Proliferation rate of degenerative NP cells in collagen I scaffold.

**DDG**	**Minimum**	**Maximum**	**Range %**	**Mean**	**SD**	**Mean difference**		**Mean fold**	**P value**
III	1048696	1116433	6.06	1081847	19182	III - V	21912	1,020	0.0014
IV	1054062	1099726	4.15	1083120	13121	III - IV	-1273	0,999	0.0014
V	1043674	1094825	4.67	1059934	13857	IV- V	23185	1,021	0.0014

Sixty three samples of degenerative IVD tissues were obtained from patients undergoing surgery due to spinal disc herniation. 4 x 10^5^ cells from each sample were grown for four weeks in collagen I scaffold. Cell proliferation data (MTT assay) were statistically analyzed on the bases of disc degeneration grade (DDG).

### Levels of catabolic, anti-catabolic and inflammatory proteins in degenerative NP cells

Three dimensional culture of 4 x 10^5^ NP cell in collagen I scaffold for four weeks demonstrated high and increasing expression levels of the catabolic factor ADAMTS-4 with mean expression values of 1697 + 169 pg/ml, 3196 + 176 pg/ml and 5884 + 175 pg/ml for degeneration grades III, IV and V respectively (P < 0.0001). It shows about 3.5 fold increased mean expression value between degeneration grades III - V. Also high but similar expression levels of ADAMTS-5 with mean expression values of 3952 + 196 pg/ml, 4150 + 141 pg/ml and 4097 + 192 pg/ml (P < 0.0025) were recorded (table 3 and Figure 2b-c).

**Table 3:** Expression levels of catabolic proteins in adult NP cells.

**Catabolic proteins**	**DDG**	**Min. pg/ml**	**Max. pg/ml**	**Range %**	**Mean pg/ml**	**SD**	**Mean difference**		**Mean fold**	**P value**
							**DDG**	**pg/ml**		
ADAMTS-4	III	1556	2283	31.84	1697	169	V - III	4186	3.466	< 0.0001
	IV	2735	3366	18.74	3196	176	V - IV	2687	1.840	< 0.0001
	V	5407	6203	12.83	5884	175	IV- III	1499	1.883	< 0.0001
ADAMTS-5	III	3600	4337	16.99	3952	196	V - III	145	1.036	< 0.0025
	IV	3914	4330	9.6	4150	141	V - IV	-52	0.987	< 0.0025
	V	3786	4390	13.75	4097	192	IV- III	197	1.050	< 0.0025
MMP-1	III	96	133	27.81	112	11.95	V - III	176	2.567	< 0.0001
	IV	243	279	12.90	260	10.25	V - IV	29	1.111	< 0.0001
	V	268	319	15.98	289	15.67	IV- III	147	2.309	< 0.0001
MMP-2	III	62	80	22.50	70	6.142	V - III	3.129	1.044	0.3295
	IV	61	79	22.78	70	7.281	V - IV	3.271	1.046	0.3295
	V	64	81	20.98	73	5.483	IV- III	-0.142	0.997	0.3295
MMP-3	III	8453	8748	3.37	8700	54,52	V - III	63	1.007	0.001
	IV	8678	8981	3.37	8835	93.39	V - IV	-72	0.991	0.001
	V	8443	8939	5.54	8763	153.4	IV- III	135	1.015	0.001
MMP-7	III	335	352	4.82	343	4.502	V - III	-119	0.651	< 0.0001
	IV	259	276	6.15	268	4.694	V - IV	-45	0.831	< 0.0001
	V	212	238	10.92	223	6.460	IV- III	-74	0.782	< 0.0001
MMP-13	III	430	462	6.92	436	7.167	V - III	6.564	1.015	0.0233
	IV	434	460	5.65	442	6.472	V - IV	1.348	1.003	0.0233
	V	432	482	10.37	443	10.66	IV- III	5.215	1.011	0.0233

MMP-8, MMP-9 and MMP-10 expression levels were below the minimum detectable dose (MDD) of our detection system. MDD of MMP-8 < 20 pg/ml, MMP-9 < 156 pg/ml and MMP-10 < 4 pg/ml.

Sixty three samples of degenerative IVD tissues were obtained from patients undergoing surgery due to spinal disc herniation. 4 x 10^5^ cells from each sample were grown for four weeks in collagen I scaffold. The protein concentration data (ELISA) of catabolic factors were statistically analyzed from 100 µg total protein extracts on the bases of disc degeneration grade (DDG).

**Figure 3:**
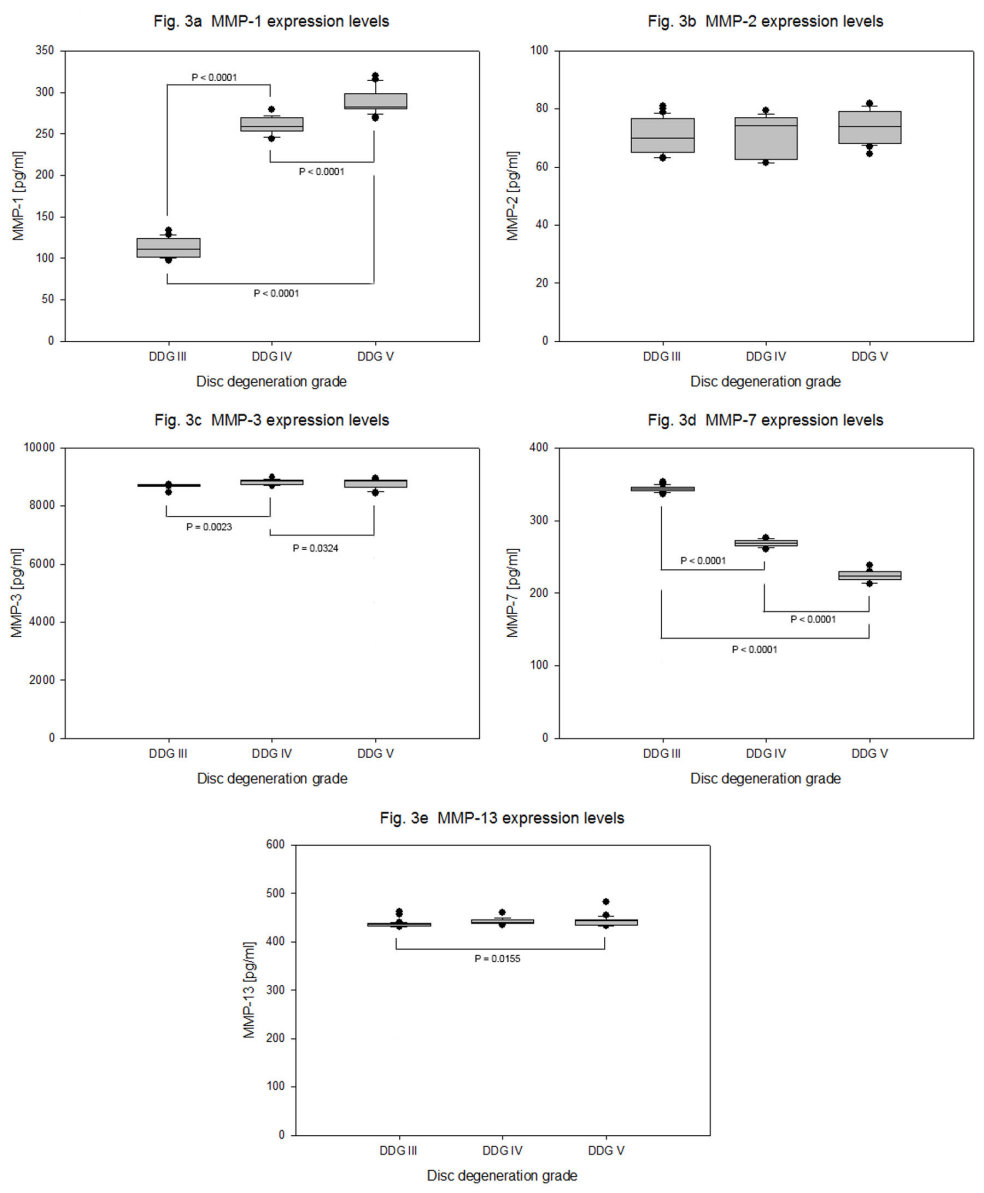
Expression levels of catabolic proteins MMPs in degenerative NP cells. NP cells were isolated from 63 samples of degenerative IVDs and 4 x 10^5^ cells from each sample were cultured for four weeks in collagen I scaffold. Concentration data (ELISA) of MMPs from 100 µg total protein extracts were statistically analyzed on the bases of disc degeneration grade (DDG). Box plots with whiskers min. to max. show MMP-1 expression levels (Figure 3a), MMP-2 expression levels (Figure 3b), MMP-3 expression levels (Figure 3c), MMP-7 expression levels (Figure 3d) and MMP-13 expression levels (Figure 3e).

Among all tested catabolic factors MMP-3 was expressed at highest levels in degeneration grade III, IV and V with similar mean expression values of 8700 + 54.52 pg/ml, 8835 + 93.39 pg/ml and 8763 + 153 pg/ml (P = 0.001) respectively. In comparison with MMP-3 expression, extremely lower and constant expression levels of MMP-13 with mean expression values 436 + 7.167 pg/ml, 442 + 6.472 pg/ml and 443 + 10.66 pg/m (P = 0.0233), and decreasing expression levels of MMP-7 with mean expression values 343 + 4.502 pg/ml, 268 + 4.694 pg/ml and 223 + 6.460 pg/m (P < 0.0001), as well as increasing expression levels of MMP-1 with mean expression values 112 + 11.95 pg/ml, 260 + 10.25 pg/ml and 289 + 15.65 pg/ml (P < 0.0001) and equivalent expression levels of MMP-2 with mean expression values less than 74 pg/ml were measured (table 3 and Figure 3). The expression levels of MMP-8, MMP-9 and MMP-10 were below the minimum detectable dose (MDD) of our detection system (MDD of MMP-8 < 20 pg/ml, MMP-9 < 156 pg/ml and MMP-10 < 4 pg/ml). 

**Figure 4:**
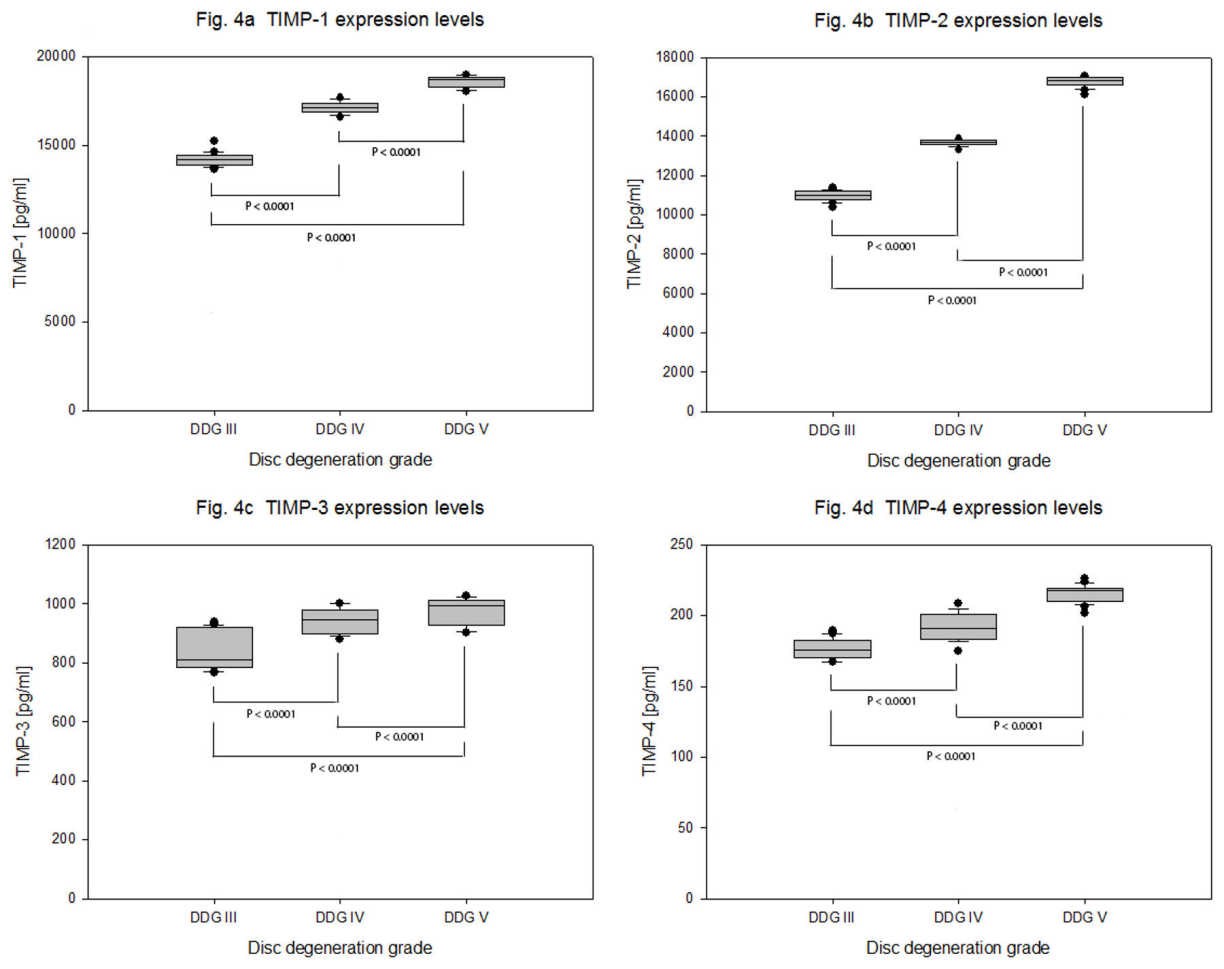
Expression levels of anti-catabolic proteins TIMPs in degenerative NP cells. NP cells were isolated from 63 samples of degenerative IVDs and 4 x 10^5^ cells from each sample were cultured for four weeks in collagen I scaffold. Concentration data (ELISA) of TIMPs from 100 µg total protein extracts were statistically analyzed on the bases of disc degeneration grade (DDG). Box plots with whiskers min. to max. show TIMP-1 expression levels (Figure 4a), TIMP-2 expression levels (Figure 4b), TIMP-3 expression levels (Figure 4c) and TIMP-4 expression levels (Figure 4d).

The high expression levels of MMP-3 were counteracted by higher and increasing expression levels of TIMP-1 and TIMP-2 with mean expression values of 14189 + 348 pg/ml, 17095 + 336 pg/ml and 18578 + 322 pg/ml for TIMP-1 (P < 0.0001) and 10966 + 248 pg/ml, 13640 + 156 pg/ml and 16757 + 264 pg/ml (P < 0.0001) for TIMP-2. In contrast, considerably lower but slightly increasing TIMP-3 expression levels were recorded with mean expression values of 843 + 67 pg/ml, 939 + 45 pg/ml and 973 + 46 pg/ml (P < 0.0001). TIMP-4 had the lowermost expression levels with mean expression values of 176 + 7.072 pg/ml, 192 + 9.963 pg/ml and 215 + 6.167 pg/ml (P < 0.0001) respectively (table 4 and Figure 4). 

**Table 4:** Expression levels of anti-catabolic and inflammatory proteins in adult NP cells.

**Anti-catabolic proteins**	**DDG**	**Min. pg/ml**	**Max. pg/ml**	**Range %**	**Mean pg/ml**	**SD**	**Mean difference**		**Mean fold**	**P value**
							**DDG**	**pg/ml**		
TIMP-1	III	13621	15187	10.31	14189	348	V - III	4388	1.309	< 0.0001
	IV	16531	17648	6.32	17095	336	V - IV	1482	1.086	< 0.0001
	V	18036	18941	4.77	18578	322	IV- III	2906	1.204	< 0.0001
TIMP-2	III	10399	11356	8.42	10966	248	V - III	5790	1.528	< 0.0001
	IV	13290	13839	3.96	13640	156	V - IV	3117	1.228	< 0.0001
	V	16103	17042	5.50	16757	264	IV- III	2673	1.243	< 0.0001
TIMP-3	III	765	936	18.26	843	67	V - III	129	1.153	< 0.0001
	IV	879	1000	12.10	939	45	V - IV	34	1.036	< 0.0001
	V	902	1028	12.25	973	46	IV- III	95	1.113	< 0.0001
TIMP-4	III	167	189	15.22	176	7.072	V - III	39	1.223	< 0.0001
	IV	174	208	24.34	192	9.963	V - IV	23	1.122	< 0.0001
	V	201	226	11.06	215	6.167	IV- III	15	1.089	< 0.0001
**Inflammatory proteins**	**DDG**	**Min. pg/ml**	**Max. pg/ml**	**Range %**	**Mean pg/ml**	**SD**	**Mean difference**		**Mean fold**	**P value**
							**DDG**	**pg/ml**		
IL-1β	III	101	113	10,61	110	3.770	V - III	29.362	1.264	< 0.0001
	IV	112	127	11.81	121	6.326	V - IV	18.315	1.150	< 0.0001
	V	132	143	7.69	140	2.895	IV- III	11.047	1.099	< 0.0001
IL-1 R	III	119	140	15.00	128	5.944	V - III	36.998	1.288	< 0.0001
	IV	133	158	15.85	146	8.229	V - IV	18.670	1.127	< 0.0001
	V	150	176	14.77	165	7.827	IV- III	18.328	1.142	< 0.0001
TNF-α	III	84	101	16.83	93	4.666	V - III	24.314	1.259	< 0.0001
	IV	97	114	14.91	105	6.259	V - IV	12.366	1.116	< 0.0001
	V	110	125	12.00	118	4.486	IV- III	11.948	1.127	< 0.0001
TNF-α R1	III	82	88	6.81	85	1.856	V - III	1.859	1.021	0.0002
	IV	83	90	7.77	87	1.796	V - IV	-0.227	0.997	0.0002
	V	84	89	5.61	86	1.426	IV- III	2.087	1.024	0.0002

Sixty three samples of degenerative IVD tissues were obtained from patients undergoing surgery due to spinal disc herniation. 4 x 10^5^ cells from each sample were grown for four weeks in collagen I scaffold. The protein concentration data (ELISA) of anti-catabolic and inflammatory factors were statistically analyzed from 100 µg total protein extracts on the bases of disc degeneration grade (DDG).

**Figure 5:**
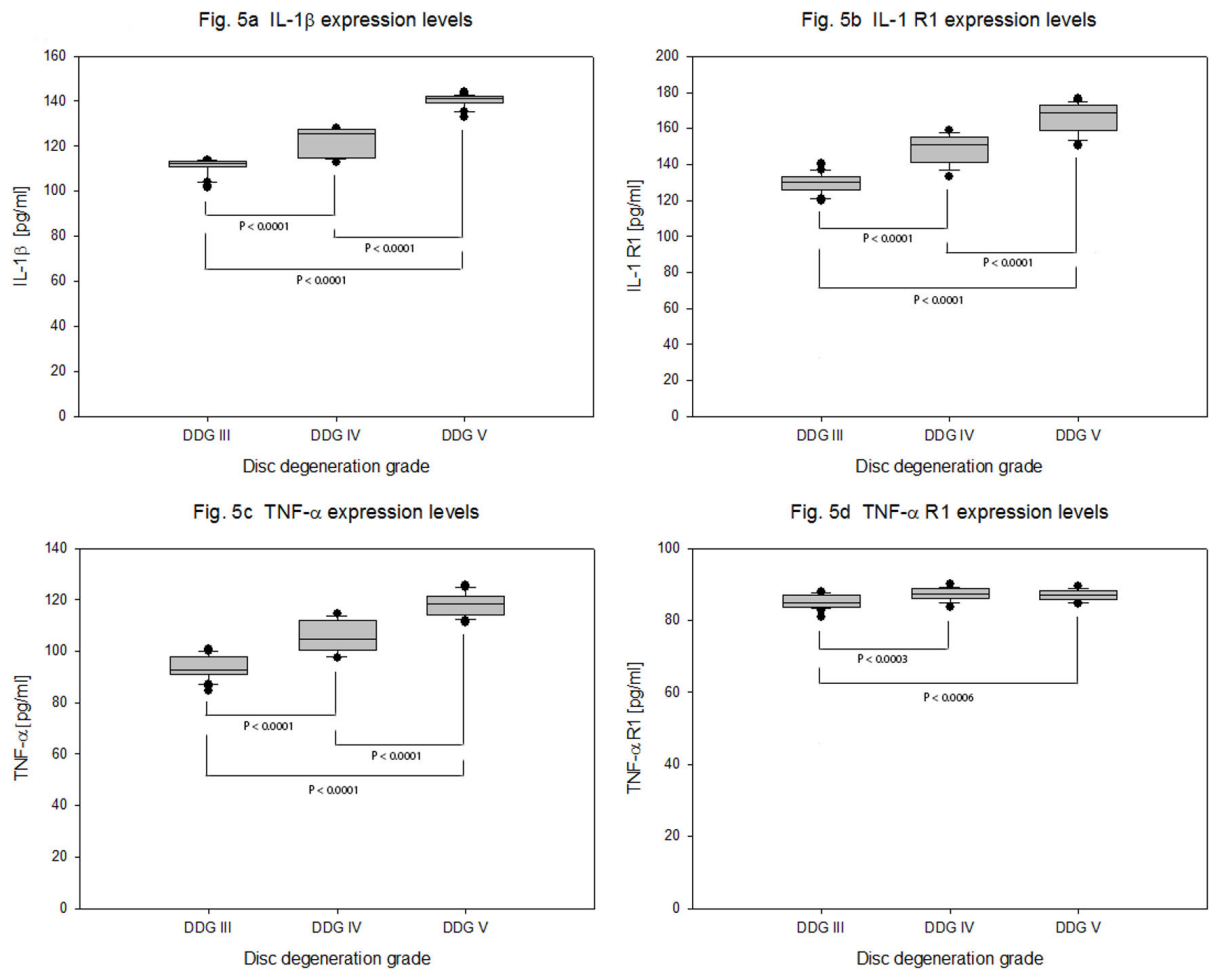
Expression levels of inflammatory cytokines in degenerative NP cells. NP cells were isolated from 63 samples of degenerative IVDs and 4 x 10^5^ cells from each sample were cultured for four weeks in collagen I scaffold. Concentration data (ELISA) of inflammatory cytokines from 100 µg total protein extracts were statistically analyzed on the bases of disc degeneration grade (DDG). Box plots with whiskers min. to max. show IL-1β expression levels (Figure 5a), IL-1 R expression levels (Figure 5b), TNF-α expression levels (Figure 5c) and TNF-α R1 expression levels (Figure 5d).

Very low expression levels of inflammatory cytokines IL-1β, IL-1 R, TNF-α and TNF-α R1 were detected in degeneration grade III, IV and V. Compared to TNF-α and TNF-α R1, higher expression levels of IL-1β and IL-1 R were recorded. The mean expression values of IL-1β were 110 + 3.770 pg/ml, 121 + 6.326 pg/ml, 140 + 2.895 pg/ml (P < 0.0001), and of IL-1 R were 128 + 5.944 pg/ml, 146 + 8.229 pg/ml, 165 + 7.827 pg/ml (P < 0.0001). The mean expression values of TNF-α were 93 + 4.666 pg/ml, 105 + 6.259 pg/ml, 118 + 4.486 pg/ml (P < 0.0001) and of TNF-α R1 were 85 + 1.856 pg/ml, 87 + 1.796 pg/ml, 86 + 1.426 pg/ml (P = 0.0002) respectively. About 30 % increased mean expression value of IL-1β, IL-1 R and TNF-α were recorded between degeneration grade III - V , whereas the mean expression values of TNF-α R1 remained constant (table 4 and Figure 5).

The calculated significance of concentration changes as a function of age by decade showed increasing expression levels of MMP-1 (P < 0.0001) and TNF-α (P < 0.0001). Gender does not appear to play a role in influencing the expression levels of the analyzed catabolic, anti-catabolic and inflammatory proteins (data not shown).

### Levels of anabolic and matrix proteins in degenerative NP cells

**Figure 6:**
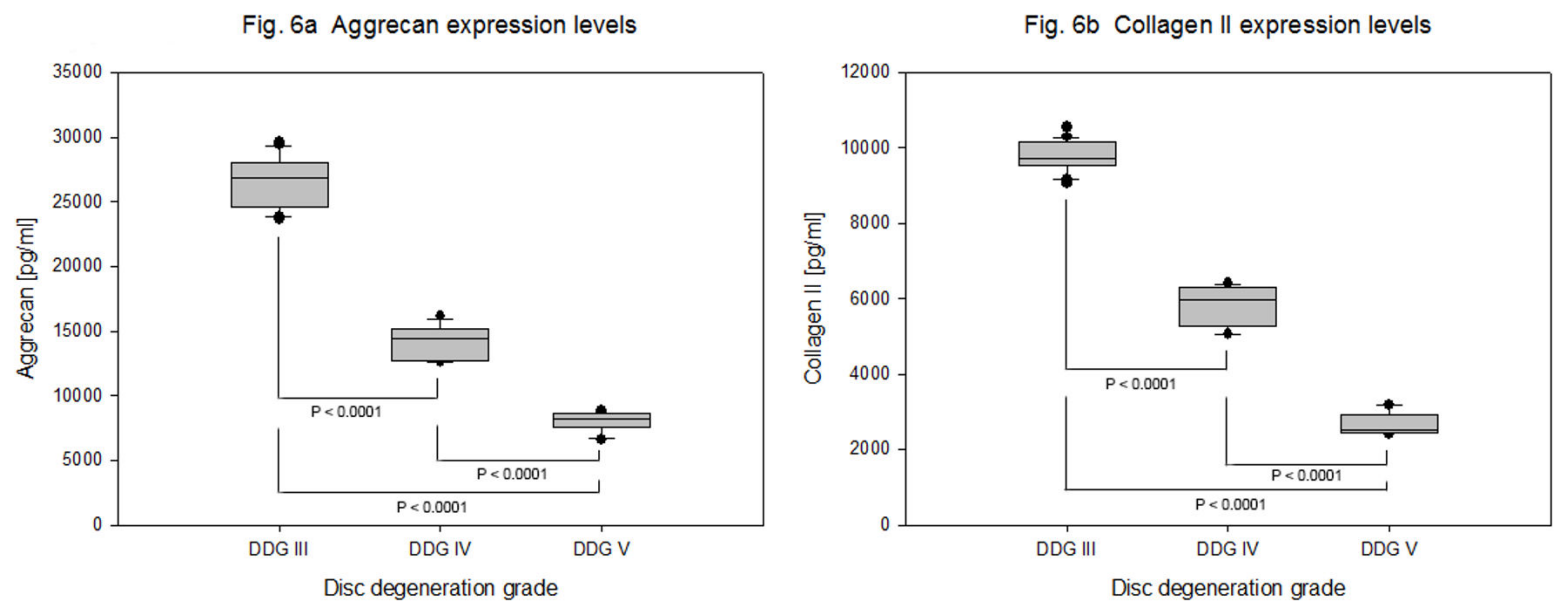
Expression levels of matrix proteins in degenerative NP cells. NP cells were isolated from 63 samples of degenerative IVDs and 4 x 10^5^ cells from each sample were cultured for four weeks in collagen I scaffold. Concentration data (ELISA) of aggrecan and collagen II from 100 µg total protein extracts were statistically analyzed on the bases of disc degeneration grade (DDG). Box plots with whiskers min. to max. show aggrecan expression levels (Figure 6a) and collagen II expression levels (Figure 6b).

Matrix proteins aggrecan and collagen II were expressed in decreasing manner with respect to degeneration grade III, IV and V. Declining expression levels of aggrecan with mean expression values of 26371 + 1978 pg/ml, 14151 + 1284 pg/ml, 7970 + 718 pg/ml (P < 0.0001) and collagen II with mean expression values of 9766 + 414 pg/ml, 5821 + 517 pg/ml, 2673 + 283 pg/ml (P < 0.0001) were determined respectively. About 3.3 fold decreased mean expression values of aggrecan and similarly about 3.7 fold decreased mean expression values of collagen II were recorded between degeneration grade III - V (table 5 and Figure 6).

**Table 5:** Expression levels of anabolic factors and matrix proteins in adult NP cells.

**Anabolic proteins**	**DDG**	**Min. pg/ml**	**Max. pg/ml**	**Range %**	**Mean pg/ml**	**SD**	**Mean difference**		**Mean fold**	**P value**
							**DDG**	**pg/ml**		
Anabolic protein expression levels were below the minimum detectable dose (MDD). MDD of BMP-2 < 11 pg/ml, BMP-4 < 1 pg/ml, BMP-6 < 3 pg/ml, BMP-7 < 2 pg/ml, IGF-1 < 25 pg/ml, TGF-β1 and TGF-β3 < 5 pg/ml.										

**Matrix proteins**	**DDG**	**Min. pg/ml**	**Max. pg/ml**	**Range %**	**Mean pg/ml**	**SD**	**Mean difference**		**Mean fold**	**P value**
							**DDG**	**pg/ml**		
Aggrecan	III	23663	29598	20.05	26371	1978	III - V	18400	3.308	< 0.0001
	IV	12556	16152	22.26	14151	1284	III - IV	12219	1.863	< 0.0001
	V	6557	8793	25.42	7970	718	IV- V	6181	1.775	< 0.0001
Collagen type II	III	9037	10566	14.47	9766	414	III – V	7092	3.652	< 0.0001
	IV	5059	6390	20.82	5821	517	III – IV	3945	1.677	< 0.0001
	V	2418	3269	26.03	2673	283	IV- V	3147	2.177	< 0.0001

Collagen I expression level was below MDD (MDD < 217 pg/ml).

Sixty three samples of degenerative IVD tissues were obtained from patients undergoing surgery due to spinal disc herniation. 4 x 10^5^ cells from each sample were grown for four weeks in collagen I scaffold. The protein concentration data (ELISA) of anabolic factors and matrix proteins were statistically analyzed from 100 µg total protein extracts on the bases of disc degeneration grade (DDG).

The protein expression level of collagen I was below the minimum detectable dose (MDD) of our detection system (MDD < 217 pg/ml). Neither control 3D culture in agarose gel nor 2D culture on TC dishes delivered additional result. Thus collagen I scaffold used for 3D culture did not inhibit collagen I expression. Although the minimum detectable dose of our detection system was extremely low for anabolic factors, the protein expression levels of all tested anabolic factors remained below the MDD. The recorded MDDs were for BMP-2 < 11 pg/ml, BMP-4 < 1 pg/ml, BMP-6 < 3 pg/ml, BMP-7 < 2 pg/ml, IGF-1 < 25 pg/ml, TGF-β1 and TGF-β3 < 5 pg/ml. The calculated significance of concentration changes as a function of age by decade showed decreasing expression levels of aggrecan (P < 0.0001) and collagen II (P < 0.0001). Gender does not appear to play a role in influencing the expression levels of the anabolic and matrix proteins (data not shown). 

## Discussion

### NP cell density and IVD degeneration

Previous studies have shown that during childhood disc specimens exhibit considerably higher cell density than during adolescence [20-24]. During growth the cell environment within the disc changes harshly, as its size increases and blood supply as well as diffusion decrease, which results in decreased concentration of glucose and oxygen [25-29]. There are conflicting reports with regard to cell densities in different grades of degeneration in adulthood. Some studies reported a decline of NP cell densities with increasing degeneration [30-32]. A recent study, however, indicated comparable NP cell densities in adult age with different grades of IVD degeneration [20]. It may indicate that progressive degenerative changes in adult NP may not be caused by loss of NP cells but rather due to unfavorable phenotypic alternation of the NP cells. Similarly, our study showed comparable NP cell proliferation rates between the degeneration grades III, IV and V (table 2, Figure 2a). Neither age nor gender appears to play a role in influencing cell proliferation potential.

In this study inclusion criteria for surgery were radiographically determined intervertebral disc herniations with nerve root compression on MRI, which correlated to primary symptoms that remained unresponsive to non-operative treatment for six weeks or demonstrated progressive neurological deterioration in the face of conservative treatment. Our findings may therefore be representative of degenerative discs from such patients.

### Imbalance between catabolic and anti-catabolic factors in degenerative IVDs

In contrast to previous findings that showed the percentage of immunohistological ADAMTS-5 positive cells correlating with the age of the patients [33], we found age to be an independent factor with regard to ADAMTS-5, which was highly expressed in all grades of degeneration. Additionally, we presented high and increasing expression levels of ADAMTS-4 with 3.5 fold amplification between degeneration grades III and V (table 3, Figure 2b-c). In human osteoarthritic cartilage, ADAMTS-4 and ADAMTS-5 have been shown to cause aggrecan degradation [34,35]. *In vitro* inhibition of ADAMTS-4 and ADAMTS-5 by siRNA in rat chondrocytes could significantly increase the aggrecan and collagen II content [36,37]. Additionally, *in vivo* inhibition of ADAMTS-5 by siRNA in a rabbit annular needle-puncture model resulted in improved MRI scores with increased signal intensity and improved histological grade scores [38]. Hence, both ADAMTS-4 and ADAMTS-5 might represent attractive targets for biological treatment of human IVD degeneration.

The expression of MMPs and their counter parts TIMPs in NP cells has been controversy discussed. On one hand, the number of immunopositive cells for MMP-1, -3, -13 and ADAMTS-4 increased with the severity of degeneration and this was accompanied by increased number of immunopositive cells for TIMP-1 and -2 but not for TIMP-3 [14]. On the other hand, the most extensive immunohistochemical staining was seen for MMP-1, -2, -3, and -9 and much less for MMP-7 and -8, and this up-regulation was paralleled by greater expression of TIMP-2 and not TIMP-1[39]. Moreover, consistent and substantial up-regulated mRNA levels of MMP-3 and MMP-8 were observed and this up-regulation was paralleled by greater expression of TIMP-1 and not TIMP-2 [15]. In another study, immunohistological staining was negative for TIMP-1 in MMP-3 positive stained surgical specimens [40]. Our protein concentration data of MMP-1, -2, -3, -7, -8, -9, -10 and -13 showed by far the highest concentration levels for MMP-3 and its levels did not increase with the severity of degeneration. The mean expression values of MMP-3 was about 20 fold of MMP-13, 30 fold of MMP-1 and 120 fold of MMP-2 (Table 3 and Figure 3). The expression values of MMP-8, -9 and -10 were below the MDD (Table 3).

Contrary to the above observations, where TIMPs were expressed at lower levels than MMP-3, we found higher expression levels of TIMP-1 and -2. The high levels of MMP-3 were counteracted by higher and increasing expression levels of TIMP-1(2.1 fold of MMP-3) and TIMP-2 (1.9 fold of MMP-3). In comparison, considerably less TIMP-3 and even much less TIMP-4 expression levels were recorded. The mean expression values of TIMP-3 and -4 were about 5.2 % and 1.2 % of TIMP-1 respectively (table 4 and Figure 4).

According to our data, repression of MMP-3 as well as ectopic expression of TIMP-3, an inhibitor of ADAMTs, would potentially be more effective to enhance the regeneration potential of NP cells. As the expression levels of TIMP-1 and -2 are higher than MMP-3, it would be more interesting to focus on their posttranslational modifications than their expression. Nonetheless, adenoviral delivery of TIMP-1 to degenerative human NP cells *in vitro* resulted in increased proteoglycan synthesis in pellet cultures [41].

### Implication of inflammatory cytokines in the pathogenesis of IVD degeneration

In non-degenerative, degenerative and herniated human disc cells the expressions of IL-1β, TNF-α and their receptors IL-1 R and TNF-α R1 were investigated using quantitative real time PCR and immunohistochemistry. Higher levels of IL-1β, TNF-α and IL-1 R expression were observed in degenerative or herniated IVDs compared to non-degenerative IVDs [16]. In another study the expression of IL-1β, IL-1 R and IL-1 Ra were shown in non-degenerative and degenerative NP and the expressions of IL-1β and IL-1 R increased with severity of degeneration [39]. In our study IL-1β and IL-1 R were expressed at higher levels than TNF-α and TNF-α R1. The protein expression levels of IL-1β, IL-1 R and TNF-α were increased with severity of degeneration. However the expression levels of TNF-α R1 remained steady throughout all degeneration grades (table 4 and Figure 5). Even though both IL-1β and TNF-α could be implicated in the pathogenesis of disc degeneration, IL-1β could be a better therapeutic target for IVD regeneration. Furthermore, treatment of human NP and AF cells with 10 ng/ml recombinant IL-1β has shown increased expression of MMP-3, MMP-13 and ADAMTS-4 as well as decreased expression of aggrecan, collagen II, collagen I, and SOX6 [42]. In addition, treatment of NP cells with TNF-α and IL-1β increased the NF-κΒ dependent expression of syndecan-4 (SDC4), which may cause ADAMTS-5 mediated degradation of aggrecan [43].

### Expression of growth factors and matrix proteins in degenerative IVDs

Age and degeneration grade related changes in the extracellular matrix of NP and AF of human IVDs have been presented [44,45]. Several growth factors such as TGF-βs, BMPs, IGF-1, bFGF, GDF-5 and PDGF have been shown to be important biological components for the stimulation of matrix synthesis [46]. Furthermore, the therapeutic potential of growth factors TGF-β1, PRP, BMP-2, BMP-12 and growth factors combination (TGF- β1, IGF-1 and BMP-2) has been discussed comprehensively [2]. However, the endogenous expression levels of growth factors in IVDs have scarcely been published. Only a few number of publications presented endogenous expression of growth factors bFGF, TGF-β1, TGF-β2 and growth factor receptors TGFβ RII, FGF R3 and BMP RI in normal and degenerative IVDs using immunohistochemical analysis [17,47]. In contrast, although we applied a large amount of total protein extract from NP cells (100 µg) and the minimum detectable doses for tested growth factors were very low (1 - 25 pg/ml), the protein expression levels of all tested growth factors (BMP-2, -4, -6, -7, IGF-1, TGF-β1 and TGF-β3) remained below the minimum detectable doses (table 5). Hence, our data underline an imbalance between the expression levels of anabolic and catabolic proteins in degenerative NP cells. Furthermore, we measured with increasing degeneration decreasing expression levels of aggrecan and collagen II. About 3.3 fold decreased mean expression values of aggrecan and 3.7 fold decreased mean expression values of collagen II were recorded between grade III - V (table 5 and Figure 6).

## Conclusions

Considering the protein concentration data of the analyzed degenerative and regenerative factors of IVD, repression of the catabolic factors ADAMTS-4, ADAMTS-5 and MMP-3 as well as the inflammatory cytokines IL-1β and IL-1 R could be a promising approach. The repression course could be combined with ectopic expression of the anabolic factors and the anti-catabolic factor TIMP-3. This combinatorial process could be a prospective objective to enhance the regeneration potential of degenerative NP cells.
